# Intraday Activity Levels May Better Reflect the Differences Between Major Depressive Disorder and Bipolar Disorder Than Average Daily Activity Levels

**DOI:** 10.3389/fpsyg.2018.02314

**Published:** 2018-12-07

**Authors:** Tsunehiko Tanaka, Kumiko Kokubo, Kazunori Iwasa, Kosuke Sawa, Naoto Yamada, Masashi Komori

**Affiliations:** ^1^Educational Psychology Course, Faculty of Education, Niigata University, Niigata, Japan; ^2^Department of Psychiatry, Shiga University of Medical Science, Ōtsu, Japan; ^3^Graduate School of Engineering, Osaka Electro-Communication University, Neyagawa, Japan; ^4^Department of Educational Psychology, Shujitsu University, Okayama, Japan; ^5^Faculty of Human Sciences, Department of Psychology, Senshu University, Kawasaki, Japan; ^6^Kamibayashi Memorial Hospital, Ichinomiya, Japan; ^7^Faculty of Information and Communication Engineering, Osaka Electro-Communication University, Neyagawa, Japan

**Keywords:** depressive disorder, bipolar disorder, intraday activity pattern, inpatients, wearable sensors

## Abstract

It is important to establish an objective index to differentiate mood disorders (i.e., bipolar disorder; BD and major depressive disorder; MDD). The present study focused on the pattern of changes of physical activity in the amount of activity intraday, and examined the relationship between activity patterns and mood disorders. One hundred and eighteen inpatients with MDD or BD in a depressive state provided the activity data by using wearable activity trackers for 3 weeks. In order to illuminate the characteristic patterns of intraday activities, Principal Component Analysis (PCA) was adopted to extract the main components of intraday activity changes. We found that some of the PCs reflected the differences between the types of mood disorder. BD participants showed high activity pattern in the morning and low activity pattern in evenings. However, MDD showed the opposite. Our results suggest that activity tracking focused on daytime activity patterns may provide objective auxiliary diagnostic information.

## Introduction

It can be challenging to diagnose major depressive disorder (MDD) on the first medical examination. Generally, clinicians diagnose mood disorders through comparing the observed symptoms and episodes with diagnostic criteria such as the Diagnostic and Statistical Manual of Mental Disorders (DSM). The symptoms associated with mood disorders are generally investigated with the use of a clinical interview. At the same time, clinicians may observe depressive symptoms or states in their patients during each medical examination. Typically, the psychiatric diagnosis of MDD is guided by this information. In this study, we focused on physical activity patterns which are potentially useful tool to help inform the diagnosis of MDD, and have the added benefit of being objectively assessed by non-invasive and easy to use equipment.

Regardless of current symptoms, obtaining a detailed history is vital to accurate diagnosis of mood disorder. For example, if a patient presents with current depressive symptoms, it is important to know whether there is a prior manic episode, as this would automatically lead to a diagnosis of bipolar disorder (BD) as opposed to MDD (American Psychiatric Association, 2013). Likewise, if manic symptoms appear following a major depressive episode, the diagnosis would change from MDD to BD. In a previous study, which surveyed participants with a diagnosis of BD, researchers found that as many as one-third were initially diagnosed with depression (Hirschfeld et al., 2003). Diagnosing a mood disorder is particularly difficult during the depressive phase, because depressive symptoms commonly appear in a variety of physical and psychiatric disorders (Kupfer et al., 2012). There are no clear differences in diagnostic criteria between depressive symptoms found in MDD and those found in BD. Therefore, at the time of the first depressive episode, it is impossible to rule out the possibility of a bipolar depression. Thus, in order to accurately diagnose mood disorders, especially BD, it is important to monitor patients over time. Giving an incorrect diagnosis will not only delay the onset of proper treatment, but, in the case of BD, administering antidepressants (e.g., Selective Serotonin Reuptake Inhibitors) to BD patients may cause “rapid cycling” (i.e., the patient oscillates between brief manic and depressive episodes) (Bowden, 2005). Therefore, early and accurate diagnosis is critical in psychiatric disorders in order to administer the most appropriate, evidence-based treatment (Valentí et al., 2015).

There is a call amongst researchers in mood disorders to investigate more objective indicators of depression or BD including potential biomarkers (Holsboer, 2008). Several studies have already reported the presence of genes that appear to be associated with depression (Holsboer, 2008; Cai et al., 2015) and the development of BD. There have additionally been some associations found between depression and the hippocampus (Sheline et al., 1996; Hori et al., 2016) and the volume of the orbitofrontal cortex (Vythilingam et al., 2002). Furthermore reduced volume and structural abnormality of white matter in patients with BD (Ballmaier et al., 2004) has also been pointed out. These studies have focused on the effects of new drugs on neurons as well as their impact on the onset of mood disorders. Such research does not, however, improve clinicians’ abilities to diagnose a mood disorder. Brain activity observed during a verbal fluency task may be an objective marker that can differentiate between MDD and BD with depression (Benedetti and Bollettini, 2014; Kinoshita et al., 2016), but consistent results have not been obtained (Nishimura et al., 2015). Thus, although many possible biomarkers have been proposed to differentiate a number of psychiatric disorders, there is still considerable research needed to establish cost-effective and clinically useful indicators that can be used to aid in accurate diagnosis.

Several studies have shown that activity level can be a potential index of mood disorders. There have been recent attempts to assess mood disorders based on the amount of physical activity exhibited by a patient (Ono et al., 2015). Traditionally MDDs and other mood disorders are closely associated with abnormal levels of activity (i.e., either an abnormal increase or decrease). It has long been known that depressed patients are characterized by diminished activity (Burton et al., 2013). For example, activity level has traditionally been viewed as an important indicator of a mood disorder amongst individuals such as Kraepelin. According to his observations, patients with depression had little movement all day, whereas others had the characteristic of getting very excited and displaying hyperactivity, which he classified as mania (Volkers, 2003). Thus, hyperactivity is a key diagnostic criterion for patients with BD (Kraepelin, 1904). Even now and indeed, level of activity is generally included in the diagnostic criteria (American Psychiatric Association, 2013).

Traditional diagnostic methods in psychiatry include patient self-report as well as behavioral observation. Furthermore, tools such as self-report questionnaires have developed and used frequently. However, in recent years it has been pointed out that these questionnaires carry a certain amount of subjectivity due to the fact that they are still based on self-report. Self-administered questionnaires and simple monitoring tables have been used to assess the degree of activity in participants (Sadock et al., 2015), however, these subjective techniques have proved problematic (Helmerhorst et al., 2012) as there have only been small correlations found between activity measured by an accelerometer and activity subjectively declared on self-report questionnaires (Saint-Maurice et al., 2014).

There have been recent attempts to assess mood disorders using activity trackers to obtain a more objective measure of the amount of activity exhibited by patients (Tully et al., 2014). Janney et al. (2014) found that level of activity provides a good diagnostic indicator of BD (Kung et al., 2015). On the other hand, research suggests that total activity levels are not necessarily always related to symptoms of mood disorders. For example, Razavi et al. (2011) examined the relationship between scores on the Hamilton Depression Rating Scale and the activity level for 10 participants with a diagnosis of MDD. The authors did not find a correlation between the severity of the depression (evaluated by the total score of the measure), though it was found to be related to the participants’ activity levels and the items related to changes in psychomotor activity.

In this study we focused on the pattern of changes in activities within a day, because it would be more useful than entire activity level for diagnosis of MD. In the DSM-5, MDD, and BD share the same criteria for the diagnosis of depression. In other words, no distinction is made between MDD and BD at least on the basis of diagnostic criteria for depressive state. However, it is also known that there are certain differences in clinical symptoms between these conditions. For example, during the depressive state of BD, hypersomnic tendency, high fatigability, and weight gain become more prominent, whereas early morning awakening is more common in MDD (Bowden, 2005). Therefore, these features could be reflected in daily activity patterns. It is well known that depression and the activity in the autonomic nervous system are related (Volkers, 2003). It is clear that individuals who tend to be nocturnal are more likely to suffer from depression (Merikanto et al., 2013). There are also reports that circadian rhythm sleep disorders, including those related to sleep-wake rhythms, often precede the worsening of symptoms in individuals with BD (Murray and Harvey, 2010), and that the circadian rhythms of individuals with BD are not stable (Jones et al., 2005). Also, changes in intraday activity levels are strongly influenced by one’s social environment (such as daily routines of work, commuting, and eating). Anhedonia, a core symptom of depression, reduces the experience of reward felt when engaging in social activities. As a result, depression may reduce one’s sensitivity to social stimuli, and it may appear in features of activity patterns within the day. So we think that it would be promising index to pay attention to the pattern of changes in activities.

In this study, inpatients diagnosed with either MDD or bipolar depression were instructed to use wearable activity trackers to measure their sequential activity levels for 3 weeks in 2-min epochs. We divided the 3-week data of each participant into one-day intervals. To summarize the differences in intraday activity changes, we extracted major intraday activity patterns by using PCA. We examined the relationship between the major components of activity changes and the diagnosis in order to see whether there were different patterns existing amongst those diagnosed with unipolar versus bipolar depression.

## Materials and Methods

### Participants

All of the procedures employed in this study were approved by the Ethics Committee of the Shiga University of Medical Science (25-135-2) and was conducted in accordance with the principles of the Declaration of Helsinki. All participants provided written informed consent prior to participating in this study. Activity data ware acquired between June 2009 and September 2013. Participants diagnosed with MDD or BD were recruited from the inpatient unit of the Department of Psychiatry, Shiga University located at the Medical Science Hospital. Participants were diagnosed by two independent psychiatrists on the basis of DSM IV-TR criteria and were excluded from the study if they were found to have organic central nervous system disorders, epilepsy, mental retardation, substance-related disorders, or severe somatic disorders. As a result, 94 inpatients provided activity data (major depression: *n* = 59, 18 male and 41 female, age 13–85 years, mean age = 53.7, *SD* = 18.6; BD with depression: *n* = 35, 23 male and 12 female, age 16–85 years, mean age = 46.9, *SD* = 20.5; participants with BD included 15, type I inpatients, 17, type 2 inpatients, and three inpatients not otherwise specified) after excluding certain participants according to the criteria described in the next section. Participants demographic and characteristics are shown in Table 1. All participants with BD were confirmed to be in a depressed state through general clinical interviews. Consensus diagnoses were made by two psychiatrists independently using DSM-IV-TR criteria.

**Table 1:** Demographic and clinical characteristics of the participants.

Variables	MDD (*n* = 57)	BD (*n* = 35)
Age (years)		
Mean	53.7	46.9
SD	18.6	20.5
Gender (n)		
Male	18	23
Female	41	12
Global assessment of functioning score		
Mean	24.4	26.8
SD	8.3	12.0
Duration of illness (years)		
Mean	6.5	6.7
SD	6.7	7.2
Medication (n)		
Antipsychotics	12	17
Mood stabilizer	14	23
Antidepressants	49	3
Hypnotics/anxiolytics	28	6
No psychotropic medication	3	4

### Activity Monitoring

In order to measure the activity of the participants, the Actiwatch accelerometer (Philips Respironics, Murrysville, PA, United States) was used. The Actiwatch is a long-term, lightweight, activity-monitoring device that can be worn on the wrist without discomfort. The Actiwatch was placed on the non-dominant wrist of each participant for several weeks.

For each 24-h period, activity was scored in 2-min epochs, resulting in 720 epochs per day. Activity data for the first three 3 weeks of consecutive epochs were adopted for each participant. If the activity was zero at all the epochs of a day, all record of the day was defined as missing values. Participants with missing values (*n* = 31) were excluded from the analyses. The 3 weeks of data were divided into days that start and end at 0:00. The total number of days of the selected data was 1,239 days for the participants with unipolar depression patients, and 735 days for the participants with bipolar depression.

### Activity Pattern Extraction

A dimensional reduction of the data space of time series is required to comprehensively examine features of intraday activity patterns. PCA is one popular dimension reduction techniques. However, there is some controversy about the validity of applying PCA to time series data, because in time series data, neighboring data points are generally correlated each other (i.e., the order or relative position of data points includes important information) that is incompatible with the assumptions of PCA. To avoid this problem, in this study, we performed a Fourier series expansion on each intraday activity sequence, and PCA was performed on the coefficients of Fourier expansion (i.e., PCA was performed in the frequency domain instead of the time domain) (Brillinger, 1981; Shumway and Stoffer, 2011). A Fourier series expansion of the functions provides a set of harmonic values that can be used to faithfully reconstruct the intraday changes in activity through an inverse Fourier series expansion: low-order coefficients describe the rough changes, whereas high-order coefficients retain information at higher frequencies. To apply this technique, it is necessary that the cycle of interest is predetermined. This study, which deals with intraday physical activity changes focused on a 24-h cycle, so that it was possible to apply this technique to the data.

The activity waveforms for each day can be regarded as finite interval functions of time *t* (*t* = 1... *T*). Through a Fourier series expansion, an activity pattern of each day is represented as the sum of trigonometric functions as follows:

(1) $$f(t)=\frac{a_{0}}{2}+\sum_{n\;=\;1}^{\infty }\left(a_{n}\cos\frac{2\pi nt}{T}+b_{n}\sin\frac{2\pi nt}{T}\right)$$

**FIGURE 1:**
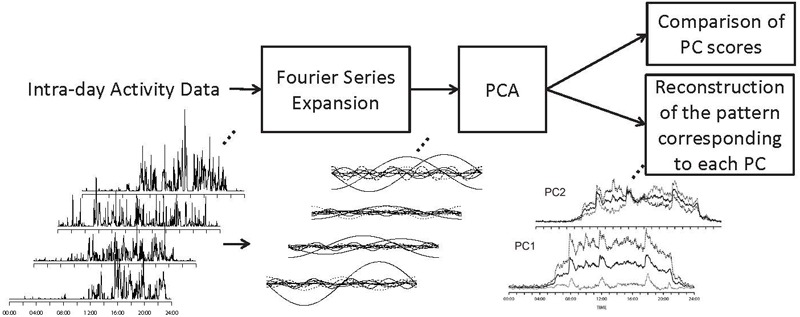
The framework of the study. Fourier series expansion was performed on each intraday activity sequence. PCA was performed on the coefficients of Fourier expansion. The waveforms corresponding to –1SD and +1SD of theoretical values along each PC were calculated based on the differences in Fourier coefficients along each PC.

Since the Fourier coefficients are a_0_, a_n_ for n : 1,…,T/2 and b_n_ for n : 1,…,T/2, a set of T + 1 coefficients are obtained through the transformation for each day and each participant. In order to analyze data with such large numbers of dimensions, a dimension reduction technique such as a PCA is applied. PCA can transform *N* dimensional data into a *k*-dimensional (k << N) principal component (PC) score, or low-dimensional representation. With these procedures, it is possible to project time-series activity data varying on many sampling points into fewer dimensional data that allows the investigation of relationships between a diagnosis and activity patterns (Figure 1).

## Results

**FIGURE 2:**
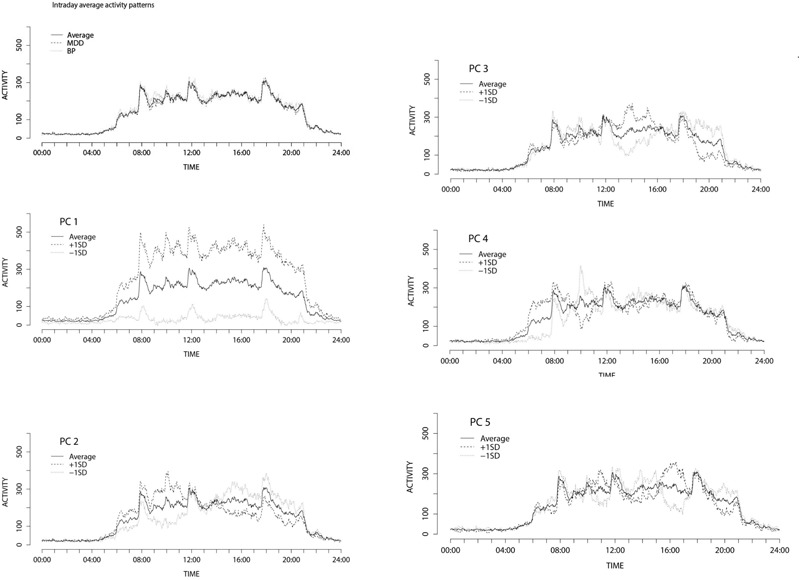
Intraday average activity patterns and Activity patterns ± 1SD variation along the principal components of intraday changes.

The average intraday activity pattern of all the participants is shown by the solid line in Figure 2 (Intraday average activity patterns). One can see that the average activity of participants rose around 6:00, which corresponds to the regular hour of rising in the inpatient unit where the data were collected. The average activity level reached its first peak around 8:00 during breakfast time. The second peak was around 10:00 corresponding to the time doctors make their rounds and regular weekday activities such as art therapy, social skill training, and recreation. The third peak from 12:00 to 12:30 occurred during lunchtime, and was followed by a relatively quiescent period. The fourth peak occurred around 18:00 corresponding to dinnertime, after which activity levels gradually decreased then fell off sharply at bedtime around 21:00. The changes in average activity level clearly correspond to the hospital schedule, which supports the fact that the data obtained from the activity trackers accurately reflect the activity of the inpatients.

Before investigating the relationship between intraday activity patterns and mood disorders, we first examined the differences between the average total daily activity levels (i.e., the sum total of time spent in all activities for a day) between the participants diagnosed with depression and participants diagnosed with BD. We performed an analysis of variance (ANOVA) on the total daily activities with diagnosis and gender as independent variables and with age as a covariate. There was no significant relationship between the diagnosis and total daily activities [*F*(1,1969) = 2.587, *p* = 0.108, η^2^ = 0.001]. There was additionally no effect of gender [*F*(1,1969) = 0.023, *p* = 0.879, η^2^ < 0.001].

There was a significant interaction between diagnosis and gender [*F*(1,1969) = 13.881, *p* < 0.001, η^2^ = 0.006]. This result suggests that total daily activity cannot be a sufficient index for clinical diagnosis of mood disorders when used alone.

On the other hand, it can be seen from Figure 2 that the average activity patterns of participants with a diagnosis of BD (dotted line) were markedly different from the average activity patterns of participants with a diagnosis of depression (dashed line), particularly around 6:00, 9:00, and 12:00, thereby suggesting that intraday activity patterns may reflect some significant differences in pathology.

We performed a Fourier series expansion on each time series of activity levels for each day up to the 360th harmonic. Consequently, a sequential pattern of activity for each day was, respectively, expressed as a set of 721 dimensional independent values. To summarize the variations contained in the Fourier coefficients, a PCA was performed on the coefficients of the Fourier series expansion. Results indicated that the accumulative contribution rate of the first 5 principal components (PCs) were relatively large (PC1, 14.1%; PC2, 2.3%; PC3, 1.8%; PC4, 1.6%; PC5, 1.5%). The accumulative contribution rate of the components was 21.3%. The scores of the derived principal components were also calculated.

To interpret each PC of the intraday fluctuation patterns, the Fourier coefficients along the first to fifth PCs (corresponding to -1SD and +1SD of the theoretical values along each PC) were calculated based on the PC loadings. By using the Fourier coefficients, the differences in the waveforms of the intraday activity patterns along each PC up to the 5th PC were obtained (Figure 2, PC1-5). The first principal component was found to be linked to the total level of activity. The higher the first principal component score is, the more frequently a participant moved. For the second principal component, the +1SD. contour peaked in the morning whereas the -1SD. contour peaked in the afternoon and evening hours, suggesting that the second principal component was related to the ratio of morning hour activity to afternoon and evening hours activity. The third principal component was related to the ratio of afternoon activity to evening activity. For the +1SD., the contour peaked around 13:30. On the other hand, the -1SD. contour showed high activity in the evening. The fourth principal component was positively linked to early morning activity. The fifth principal component was positively related to activity around 16:00. The first and the second PCs were found to be related to entire features of intraday activities, while the other components were linked to relatively local features.

In order to investigate the differences between intraday activity patterns amongst participants with diagnoses of depression and BD, the effects of pathology on the principal component scores were examined. We conducted a multivariate analysis of covariance (MANCOVA) on the principal component scores up to the 5th principal component with diagnosis and gender as independent variables and age as the covariate. It was found that the principal component scores were significantly associated with diagnosis [Wilks’s lambda = 0.989, *F*(5,1965) = 4.55, *p* < 0.001, η^2^ = 0.011], gender [Wilks’s lambda = 0.993, *F*(5,1965) = 4.55, *p* = 0.014, η^2^ = 0.007], and age [Wilks’s lambda = 0.912, *F*(5,1965) = 38.11, *p* < 0.001, η^2^ = 0.088]. The interaction between diagnosis and gender was also significant [Wilks’s lambda = 0.986, *F*(5,1965) = 5.49, *p* = < 0.001, η^2^ = 0.014]. These results suggest that activity patterns may differentiate the different types of mood disorder as well as gender and age.

**FIGURE 3:**
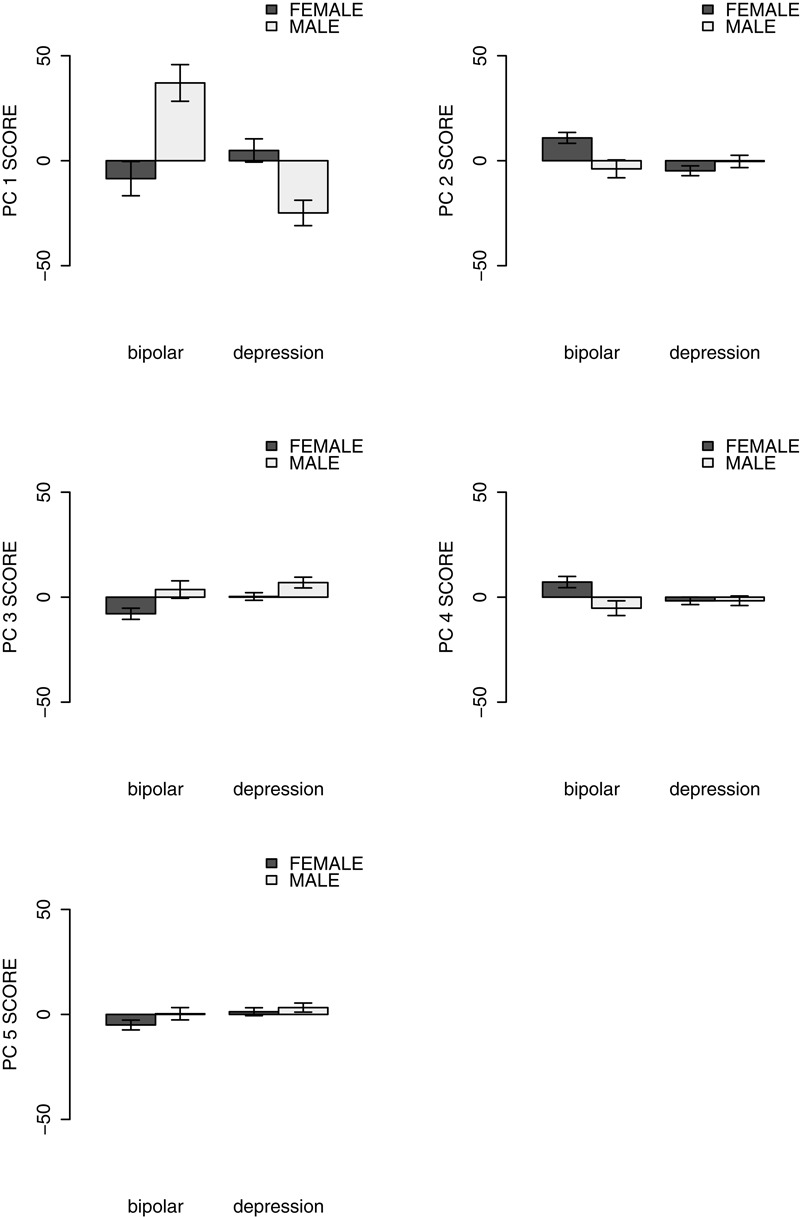
Mean PC scores of major depression and bipolar disorder.

Based on the outcome of the MANOVA, each dependent variable (from the 1st to the 5th principal component scores of the set of coefficients) was then examined using a univariate two-way analysis of covariance (ANCOVA) with diagnosis and gender as the independent variables and with age as a covariate. Figure 3 shows the relationship between diagnosis and the average principal component score for each component.

The main effect of the pathology group on the 1st PC score was insignificant [*F*(1,1969) = 2.022, *p* = 0.155, η^2^ = 0.001]. According to Figure 3, the 1st PC was related to the total level of activity. This suggests that there was no clear relationship between the total level of the activity and the clinical diagnosis. There was a significant relationship between the 2nd PC scores and the pathology group [*F*(1,1969) = 6.151, *p* = 0.013, η^2^ = 0.003], suggesting that BD was characterized by morning hyperactivity while depression was characterized by morning hypoactivity. The 3rd PC was also significantly related to the difference in pathology [*F*(1,1969) = 4.070, *p* = 0.044, η^2^ = 0.002]. For this component, BD was correlated with higher activity in the evening, whereas depression disorder was positively correlated with activity in the afternoon. For the 4th PC that reflected differences in early morning activity, the effect of the pathology group was also significant [*F*(1,1969) = 5.154, *p* = 0.023, η^2^ = 0.003], thus indicating that low activity in the early morning is clearly linked to depressive disorder. The fifth principal component was mainly related to high levels of activity around 16:00 – that is during visiting hours – and the component was positively related to depression [*F*(1,1969) = 5.232, *p* = 0.022, η^2^ = 0.003]. Moreover, the interactions between pathology groups and gender were significant for PC1 and PC2 [PC1: *F*(1,1969) = 15.236, *p* < 0.001, η^2^ = 0.008; PC2: *F*(1,1969) = 7.176, *p* = 0.007, η^2^ = 0.004; PC3: *F*(1,1969) = 0.824, *p* = 0.364, η^2^ < 0.001; PC4: *F*(1,1969) = 2.668, *p* = 0.103, η^2^ = 0.001; PC5: *F*(1,1969) = 0.146, *p* = 0.703, η^2^ < 0.001].

To confirm whether the difference in BD subtypes affected activity patterns, we conducted a two-way ANCOVA on the PC scores of BD patients with the subtype (type I and type II) and gender as the independent variables and with age as a covariate, for each PC. type I was associated with lower levels of the 1st PC score and higher levels of the 5th PC scores than type II [PC1: *F*(1,667) = 36.971, *p* < 0.001, η^2^ = 0.053; PC2: *F*(1,667) = 0.717, *p* = 0.397, η^2^ = 0.001; PC3 *F*(1,667) = 1.010, *p* = 0.315, η^2^ = 0.002; PC4: *F*(1,667) = 0.499, *p* = 0.480, η^2^ = 0.001; PC5: *F*(1,667) = 13.247, *p* < 0.001, η^2^ = 0.019]. No other significant main effect of BD subtypes on PC scores was observed. The interactive effect between BD subtype and gender was found in PC1 [PC1: *F*(1,667) = 8.712, *p* = 0.003, η^2^ = 0.013; PC2: *F*(1,667) = 0.593, *p* = 0.442, η^2^ = 0.001; PC3: *F*(1,667) = 0.126, *p* = 0.723, η^2^ = 0.000; PC4: *F*(1,667) = 0.998, *p* = 0.318, η^2^ = 0.001; PC5: *F*(1,667) = 2.735, *p* = 0.099, η^2^ = 0.004]. These results suggest that BD subtype might also be linked to differences in intraday activity patterns.

## Discussion

The results demonstrated that several temporal patterns of intraday activities are associated with the differences between unipolar depression and bipolar depression. Using wearable activity trackers, we measured the intraday fluctuations of activity levels of participants with a diagnosed mood disorder who had been admitted to a psychiatric hospital. To describe intraday activity pattern differences with a small number of variables, the intraday activity level sequences were analyzed with a combination of a Fourier series expansion and PCA. Each of the five dimensions extracted represents the typical differences in intraday activity patterns, although their activities were impacted by the different clinical diagnoses; the first PC was related to the total amount of activity occurring in each day, whereas the second PC was linked to the ratio of the morning activities to the afternoon and evening activities. The third to the fifth PCs were related to the activity in the evening, early morning and late afternoon, respectively. Among them, the second to fifth PCs were found to be related the difference in the diagnosis of mood disorders.

There was no significant difference between the first PC scores of BD depressive state and that of MDD. The first PC was found to be related to the daily total activity levels according to Figure 2 (Intraday average activity patterns), suggesting that there was no clear relationship between the total activity levels and the diagnosis. This finding is supported by the result that the daily average activity levels of participants with a diagnosis of bipolar depression were not significantly different from those of MDD. However, there was a significant difference in the first PC score between BD I and BD II. Moreover, both the first PC scores and the average activity levels were dependent on the interactions between gender and diagnosis. These results indicate complex relationships between various mood disorders and daily total activity levels. Total activity levels cannot be an index to distinguish between bipolar depression and MDD, at least within a hospital environment in which the daily lives of inpatients (e.g., going out and bedtime), are strictly controlled.

On the other hand, unlike the total daily activity, the ratio of morning to afternoon activity level was shown to be related to the difference between MDD and BD based on the results of the second PC. While the participants with MDD appeared to demonstrate a lower activity level in the morning and a rising activity level in the afternoon, the participants with bipolar depression demonstrated higher activity levels in the morning and lower activity levels in the evening. These results are consistent with a study (Monteleone and Maj, 2008) that has reported that typical unipolar depression has a characteristic malaise and activity level reduction in the morning and that these tendencies appear more strongly in female patients. There was an interactive effect of gender and diagnosis on the second PC score. This may reflect the fact that participants with bipolar depression differed in their symptom presentations depending on gender, as previous research has shown based on interviewing (Bhattacharya et al., 2011).

It was found from the comparison of the third PC scores that the participants with BD had relatively higher activity levels during the night from 19:00 to 22:00 than participants with a diagnosis of MDD, whereas patients with BD showed relatively lower activity during the daytime (13:00 to 15:00). Our results are consistent with the results of a polysomnography study (Nofzinger et al., 1991) and consultation interview study (Benazzi, 2009) that both found a decrease in the need for sleep amongst individuals with BD. It should be noted that there was no clear difference between the activity levels amongst those diagnosed with BD and MDD from 22:00 to 6:00 as these were fixed sleeping hours within the hospital. This may be due to the fact that the sleeping hours of the inpatients were controlled in several cases through the use of medication.

The association between MDD and low activity level in the morning was shown from the result of the fourth PC in addition to the second PC. The fourth PC was found to be linked to activity levels in the early morning (from 500 to 700), indicating that participants with MDD tended to have lower activity levels in the early morning. Several studies have shown that individuals with a melancholic-type MDD demonstrate a strong mood deterioration in the early morning (Stallone et al., 1973; Bouhuys, 1991; Murray, 2007). Thus the low activities of the participants with MDD in the early morning hours may have been related to a melancholic-type of MDD.

The results of this study suggest that activity tracking can provide objective auxiliary diagnostic information for mood disorders by focusing on intraday activity patterns. Activity tracking devices have the advantage of being non-invasive and highly portable, enabling continuous observation of patients with mood disorders. Thus, the activity monitoring of patients may enable real-time assessment of pathology as well as assessment of effects of antidepressant medications such as SSRIs. Furthermore, it may elucidate the process that occurs from the onset to the remission of each mood disorder. In the future, it would be necessary to verify whether the relationships between the activity patterns and each mood disorder are robust based on the activities of individuals with diagnosed mood disorder patients other than inpatients.

There are certain limitations to this study: Firstly, the analyzed data were obtained from hospitalized patients whose activity is restricted. Therefore, it was not clear whether similar activity patterns would be observed even among outpatients with the ability to freely conduct activities. Moreover, we could not investigate whether activity patterns of hospitalized patients were abnormal compared to those of healthy people because there was no healthy control group in this study. However, it is difficult to hospitalize healthy people to be a control group for comparing the results of hospitalized patient. The main aim of the current research was to clarify differences in activity patterns between BD and MDD groups, and a different pattern of activity seen in hospitalized situations, which is well-controlled, might more closely reflect characteristics of the disease itself. Another limitation is that the ratio of men to women in our study was different. The prevalence rate of MDD is 1.5 to 3 times higher in females compared to males (American Psychiatric Association, 2013), and it seems that it affects the ratio of male to female of this subject this time. The biased ratio of participants’ gender reflects gender differences in the prevalence of MDDs. Therefore, such a bias unavoidable for an observational study such as this.

In addition, this study did not take the relationship between the severity of depressive symptoms and the activity level into consideration. Symptoms of serious depression might affect the degree of activity. It is suggested that future research use a scale to assess symptoms. Similarly, it is possible that anxiety symptoms could also have an effect on the amount of activity. However, the presence and severity of comorbid anxiety were not investigated in this study. A comprehensive assessment would contribute to clarify the association with physical activity.

The cumulative contribution ratio up to the fifth PC was relatively low (21.3%). This might be partially due to the methodology of our approach. Though our method could identify global activity patterns from considerably noisy activity data obtained from the activity trackers, the microscopic structure of activity levels might have been ignored. Krane-Gartiser et al. (2014, 2015) have pointed out that activity changes occurring over a relatively short time range are associated with symptoms of mood disorders. Therefore, we cannot rule out the possibility that either such micro fluctuations or certain higher order components (above the sixth PC) could be linked to differences between MDD and BD.

It should be noted that multiple independent dimensions of activity patterns were found to be related to each mood disorder. This suggests that multiple mechanisms are responsible for the activity pattern differences between MDD and bipolar depression. It has been pointed out that several subtypes may exist in both MDD and BD (e.g., BD type I and type II). It is possible that each independent component is corresponding to such a subtype of pathology, respectively. In that sense, the difference between BD I and BD II in PC5 that was found through additional analyses could be interpreted as a feature that distinguishes both subtypes. Future research will need to consider which subtype of mood disorder is associated with which activity pattern and may make it possible to create a detailed classification of mood disorders where the symptoms of each subtype of depression can correspond with adequate treatments such as medications.

## Author Contributions

TT, MK, KI, and KS conceived and designed the study. MK and KK analyzed and interpreted the data. TT, MK, and KK collected and assembled the data. TT and MK drafted the article. TT, MK, KI, KS, and NY reviewed and edited the article. MK and NY approved the final article.

## Conflict of Interest Statement

The authors declare that the research was conducted in the absence of any commercial or financial relationships that could be construed as a potential conflict of interest.

## References

[B1] American Psychiatric Association (2013). *Diagnostic and Statistical Manual of Mental Disorders (DSM-5)^®^.* Arlington, TX: American Psychiatric Pub.

[B2] Ballmaier M. Toga A. W. Blanton R. E. Sowell E. R. Lavretsky H. Peterson J. (2004). Anterior Cingulate, Gyrus Rectus, and Orbitofrontal abnormalities in elderly depressed patients: An MRI-based Parcellation of the prefrontal cortex. *Am. J. Psychiatry* 161 99–108. 10.1176/appi.ajp.161.1.99 14702257

[B3] Benazzi F. (2009). A prediction rule for diagnosing hypomania. *Prog. Neuro Psychopharmacol. Biol. Psychiatry* 33 317–322. 10.1016/j.pnpbp.2008.12.007 19141309

[B4] Benedetti F. Bollettini I. (2014). Recent findings on the role of white matter pathology in bipolar disorder. *Harv. Rev. Psychiatry* 22 338–341. 10.1097/HRP.0000000000000007 25377606

[B5] Bhattacharya A. Khess C. R. J. Munda S. K. Bakhla A. K. Praharaj S. K. Kumar M. (2011). Sex difference in symptomatology of manic episode. *Compr. Psychiatry* 52 288–292. 10.1016/j.comppsych.2010.06.010 21497223

[B6] Bouhuys A. L. (1991). Towards a model of mood responses to sleep deprivation in depressed patients. *Biol. Psychiatry* 29 600–612. 10.1016/0006-3223(91)90095-4 1829009

[B7] Bowden C. L. (2005). A different depression: clinical distinctions between bipolar and unipolar depression. *J. Affect. Disord.* 84 117–125. 10.1016/S0165-0327(03)00194-0 15708408

[B8] Brillinger D. R. (1981). *Time Series: Data Analysis and Theory.* Philadelphia, PA: Society for industrial and applied mathematics.

[B9] Burton C. McKinstry B. Szentagotai Tǎtar A. Serrano-Blanco A. Pagliari C. Wolters M. (2013). Activity monitoring in patients with depression: a systematic review. *J. Affect. Disord.* 145 21–28. 10.1016/j.jad.2012.07.001 22868056

[B10] Cai N. Bigdeli T. B. Kretzschmar W. Li Y. Liang J. Song L. (2015). Sparse whole-genome sequencing identifies two loci for major depressive disorder. *Nature* 523 588–591. 10.1038/nature14659 26176920 PMC4522619

[B11] Helmerhorst H. J. Brage S. Warren J. Besson H. Ekelund U. (2012). A systematic review of reliability and objective criterion-related validity of physical activity questionnaires. *Int. J. Behav. Nutr. Phys. Act.* 9:103. 10.1186/1479-5868-9-103 22938557 PMC3492158

[B12] Hirschfeld R. M. A. Calabrese J. R. Weissman M. M. Reed M. Davies M. A. Frye M. A. (2003). Screening for bipolar disorder in the community. *J. Clin. Psychiatry* 64 53–59. 10.4088/jcp.v64n0111 12590624

[B13] Holsboer F. (2008). How can we realize the promise of personalized antidepressant medicines? *Nat. Rev. Neurosci.* 9 638–646. 10.1038/nrn2453 18628772

[B14] Hori H. Sasayama D. Teraishi T. Yamamoto N. Nakamura S. Ota M. (2016). Blood-based gene expression signatures of medication-free outpatients with major depressive disorder: integrative genome-wide and candidate gene analyses. *Sci. Rep.* 6:18776. 10.1038/srep18776 26728011 PMC4700430

[B15] Janney C. A. Fagiolini A. Swartz H. A. Jakicic J. M. Holleman R. G. Richardson C. R. (2014). Are adults with bipolar disorder active? Objectively measured physical activity and sedentary behavior using accelerometry. *J. Affect. Disord.* 15 498–504. 10.1016/j.jad.2013.09.009 24095103 PMC3905833

[B16] Jones S. H. Hare D. J. Evershed K. (2005). Actigraphic assessment of circadian activity and sleep patterns in bipolar disorder. *Bipolar Disord.* 7 176–186. 10.1111/j.1399-5618.2005.00187.x 15762859

[B17] Kinoshita S. Kanazawa T. Kikuyama H. Yoneda H. (2016). Clinical application of DEX/CRH test and multi-channel NIRS in patients with depression. *Behav. Brain Funct.* 12:25. 10.1186/s12993-016-0108-x 27582123 PMC5007847

[B18] Kraepelin E. (1904). *Lectures on Clinical Psychiatry.* Quebec: William Wood.

[B19] Krane-Gartiser K. Henriksen T. E. G. Morken G. Vaaler A. Fasmer O. B. (2014). Actigraphic assessment of motor activity in acutely admitted inpatients with bipolar disorder. *PLoS One* 9:e89574. 10.1371/journal.pone.0089574 24586883 PMC3930750

[B20] Krane-Gartiser K. Henriksen T. E. G. Vaaler A. E. Fasmer O. B. Morken G. (2015). Actigraphically assessed activity in unipolar depression: a comparison of inpatients with and without motor retardation. *J. Clin. Psychiatry* 76 1181–1187. 10.4088/JCP.14m09106 26214574

[B21] Kung P.-Y. Chou K.-R. Lin K.-C. Hsu H.-W. Chung M.-H. (2015). Sleep disturbances in patients with major depressive disorder: incongruence between sleep log and actigraphy. *Arch. Psychiatr. Nurs.* 29 39–42. 10.1016/j.apnu.2014.09.006 25634873

[B22] Kupfer D. J. Frank E. Phillips M. L. (2012). Major depressive disorder: new clinical, neurobiological, and treatment perspectives. *Lancet* 379 1045–1055. 10.1016/S0140-6736(11)60602-8 22189047 PMC3397431

[B23] Merikanto I. Lahti T. Kronholm E. Peltonen M. Laatikainen T. Vartiainen E. (2013). Evening types are prone to depression. *Chronobiol. Int.* 30 719–725. 10.3109/07420528.2013.784770 23688117

[B24] Monteleone P. Maj M. (2008). The circadian basis of mood disorders: recent developments and treatment implications. *Eur. Neuropsychopharmacol.* 18 701–711. 10.1016/j.euroneuro.2008.06.007 18662865

[B25] Murray G. (2007). Diurnal mood variation in depression: a signal of disturbed circadian function? *J. Affect. Disord.* 102 47–53. 10.1016/j.jad.2006.12.001 17239958

[B26] Murray G. Harvey A. (2010). Circadian rhythms and sleep in bipolar disorder. *Bipolar Disord.* 12 459–472. 10.1111/j.1399-5618.2010.00843.x 20712747

[B27] Nishimura Y. Takahashi K. Ohtani T. Ikeda-Sugita R. Okada N. Kasai K. (2015). Social function and frontopolar activation during a cognitive task in patients with bipolar disorder. *Neuropsychobiology* 72 81–90. 10.1159/000437431 26509704

[B28] Nofzinger E. A. Thase M. E. Reynolds C. F. Himmelhoch J. M. Mallinger A. Houck P. (1991). Hypersomnia in bipolar depression: a comparison with narcolepsy using the multiple sleep latency test. *Am. J. Psychiatry* 148 1177–1181. 10.1176/ajp.148.9.1177 1882995

[B29] Ono Y. Kikuchi M. Hirosawa T. Hino S. Nagasawa T. Hashimoto T. (2015). Reduced prefrontal activation during performance of the Iowa gambling task in patients with bipolar disorder. *Psychiatry Res. Neuroimaging* 233 1–8. 10.1016/j.pscychresns.2015.04.003 25978934

[B30] Razavi N. Horn H. Koschorke P. Hügli S. Höfle O. Müller T. (2011). Measuring motor activity in major depression: the association between the hamilton depression rating scale and actigraphy. *Psychiatry Res.* 190 212–216. 10.1016/j.psychres.2011.05.028 21663976

[B31] Sadock B. J. Sadock V. A. Ruiz P. (2015). *Kaplan & Sadock’s Synopsis of Psychiatry: Behavioral Sciences, Clinical Psychiatry* 11th Edn. Philadelphia, PA: Williams & Wilkins 1169–1181.

[B32] Saint-Maurice P. F. Welk G. J. Beyler N. K. Bartee R. T. Heelan K. A. (2014). Calibration of self-report tools for physical activity research: the Physical Activity Questionnaire (PAQ). *BMC Public Health* 14:461. 10.1186/1471-2458-14-461 24886625 PMC4055223

[B33] Sheline Y. I. Wang P. W. Gado M. H. Csernansky J. G. Vannier M. W. (1996). Hippocampal atrophy in recurrent major depression. *Proc. Natl. Acad. Sci. U.S.A.* 93 3908–3913. 8632988 10.1073/pnas.93.9.3908 PMC39458

[B34] Shumway R. H. Stoffer D. S. (2011). *Time Series Analysis and Its Applications* 3rd ed. New York, NY: Springer.

[B35] Stallone F. Huba G. J. Lawlor W. G. Fieve R. R. (1973). Longitudinal studies of diurnal variations in depression: a sample of 643 patient days. *Br. J. Psychiatry* 122 311–318. 10.1192/bjp.123.3.311 4746109

[B36] Tully M. A. Panter J. Ogilvie D. (2014). Individual characteristics associated with mismatches between self-reported and accelerometer-measured physical activity. *PLoS One* 9:e99636. 10.1371/journal.pone.0099636 24919185 PMC4053373

[B37] Valentí M. Pacchiarotti I. Undurraga J. Bonnín C. M. Popovic D. Goikolea J. M. (2015). Risk factors for rapid cycling in bipolar disorder. *Bipolar Disord.* 17 549–559. 10.1111/bdi.12288 25682854

[B38] Volkers A. (2003). Motor activity and autonomic cardiac functioning in major depressive disorder. *J. Affect. Disord.* 76 23–30. 10.1016/S0165-0327(02)00066-6 12943930

[B39] Vythilingam M. Heim C. Newport J. Miller A. H. Anderson E. Bronen R. (2002). Childhood trauma associated with smaller hippocampal volume in women with major depression. *Am. J. Psychiatry* 159 2072–2080. 10.1176/appi.ajp.159.12.2072 12450959 PMC3230324

